# Can We Spare the Pancreas and Other Abdominal Organs at Risk? A Comparison of Conformal Radiotherapy, Helical Tomotherapy and Proton Beam Therapy in Pediatric Irradiation

**DOI:** 10.1371/journal.pone.0164643

**Published:** 2016-10-20

**Authors:** Emmanuel Jouglar, Antoine Wagner, Grégory Delpon, Loïc Campion, Philippe Meingan, Valérie Bernier, Charlotte Demoor-Goldschmidt, Marc-André Mahé, Thomas Lacornerie, Stéphane Supiot

**Affiliations:** 1 Department of Radiation Oncology, Institut de Cancérologie de l’Ouest, boulevard Jacques Monod, Saint Herblain, 44800 France; 2 Department of Medical Physics, Centre Oscar Lambret, 3 rue Frédéric Combemale, Lille, 59000 France; 3 Department of Medical Physics, Institut de Cancérologie de l’Ouest, boulevard Jacques Monod, Saint Herblain, 44800 France; 4 Department of Statistics, Institut de Cancérologie de l’Ouest, boulevard Jacques Monod, Saint-Herblain, 44800 France; 5 Department of Radiology, Institut de Cancérologie de l’Ouest, boulevard Jacques Monod, Saint-Herblain, 44800 France; 6 Department of Radiation Oncology, Institut de Cancérologie de Lorraine, 6 Avenue de Bourgogne, Vandœuvre-lès-Nancy, 54519 France; ENEA Centro Ricerche Casaccia, ITALY

## Abstract

**Objectives:**

Late abdominal irradiation toxicity during childhood included renal damage, hepatic toxicity and secondary diabetes mellitus. We compared the potential of conformal radiotherapy (CRT), helical tomotherapy (HT) and proton beam therapy (PBT) to spare the abdominal organs at risk (pancreas, kidneys and liver- OAR) in children undergoing abdominal irradiation.

**Methods:**

We selected children with abdominal tumors who received more than 10 Gy to the abdomen. Treatment plans were calculated in order to keep the dose to abdominal OAR as low as possible while maintaining the same planned target volume (PTV) coverage. Dosimetric values were compared using the Wilcoxon signed-rank test.

**Results:**

The dose distribution of 20 clinical cases with a median age of 8 years (range 1–14) were calculated with different doses to the PTV: 5 medulloblastomas (36 Gy), 3 left-sided and 2 right-sided nephroblastomas (14.4 Gy to the tumor + 10.8 Gy boost to para-aortic lymphnodes), 1 left-sided and 4 right-sided or midline neuroblastomas (21 Gy) and 5 Hodgkin lymphomas (19.8 Gy to the para-aortic lymphnodes and spleen). HT significantly reduced the mean dose to the whole pancreas (WP), the pancreatic tail (PT) and to the ipsilateral kidney compared to CRT. PBT reduced the mean dose to the WP and PT compared to both CRT and HT especially in midline and right-sided tumors. PBT decreased the mean dose to the ispilateral kidney but also to the contralateral kidney and the liver compared to CRT. Low dose to normal tissue was similar or increased with HT whereas integral dose and the volume of normal tissue receiving at least 5 and 10 Gy were reduced with PBT compared to CRT and HT.

**Conclusion:**

In children undergoing abdominal irradiation therapy, proton beam therapy reduces the dose to abdominal OAR while sparing normal tissue by limiting low dose irradiation.

## Introduction

Thanks to combined modality treatments involving surgery, chemotherapy and radiotherapy (RT), the survival rates of childhood cancers have increased and cure rates now exceed 50% in patients with malignant solid tumor [1]. However, this high survival rate is tempered by the increasing incidence of late secondary effects of treatment, which include musculoskeletal impairment, cardio-vascular toxicity, reproductive issues, renal dysfunction, neurological impairment, and secondary malignant neoplasms [2].

Radiation-induced liver and kidney toxicity in children has been reported in several series [3]. Liver toxicity is related to the liver volume irradiated and dose received. Dose response of radiation-induced nephrotoxicity is less clear. Nevertheless kidney sparing is a key challenge especially in children with nephroblastoma because the risk of impaired renal function is increased by the use of nephrotoxic chemotherapeutic agents, surgical removal and radiotherapy. More recently, De Vathaire et al. showed that irradiation of the pancreas and particularly the pancreatic tail is associated with an increased risk of diabetes mellitus (DM) with an exponential dose response model [4]. The cumulative incidence of DM at the age of 45 was 2.3% (95% CI 0.8–6.4) in patients who had not received radiation therapy (RT) and 6.6% (4.8–9.0) in those who had (p = 0.0003). The cumulative incidence of diabetes by age 45 years for patients who had received more that 10 Gy to the tail where the islets of Langerhans are concentrated reached 16.3% and the relative risk of DM was 11.5 (95% CI 3.9–34). Likewise, irradiation with ≥ 36 Gy to the para-aortic lymph nodes was shown to increase the risk of DM in Hodgkin lymphoma survivors (2.58-fold increased standardized incidence ratio compared with general population) with an even greater risk associated with splenic irradiation [5]. The risk of DM significantly increased with higher mean radiation doses to the pancreatic tail. High dose irradiation to the pancreatic tail also increases the risk of DM in adult patients [5]. These findings suggest that the pancreas (and pancreatic tail) should also be considered to be an organ at risk (OAR) during radiotherapy planning in children.

Recent management of pediatric radiotherapy aims not only to increase cancer cure rates, but also to reduce the adverse sequelae of treatment. One strategy that could reduce RT toxicity is the use of advanced RT techniques: intensity modulated radiation therapy (IMRT) and proton beam therapy (PBT) [6–9]. IMRT is commonly used for adult cancers, and its use in pediatric cancer is increasing especially where the PTV has a complex shape, and in neurological and head and neck tumors [8]. In comparison with conventional IMRT, helical tomotherapy (HT) delivers highly conformal dose distribution potentially to a larger field with a great sparing of OAR [10–12]. However, the benefits of HT over conventional IMRT are still being investigated. PBT enables better or at least comparable dose conformation compared to IMRT especially when using intensity modulated PBT [13]. While IMRT has the disadvantage of increasing low-dose bath around target volume, PBT with its specific dose distribution profile is expected to decrease late side effects of RT and may reduce the risk of second cancer [14]. PBT is commonly used in children with craniopharyngioma, medulloblastoma, ependymoma, Ewing sarcoma, and is increasing in neuroblastoma and Hodgkin lymphoma [8, 9]. We present the result of a feasibility study that compared the potential of conformal radiotherapy (CRT), HT and PBT to spare the abdominal organs at risk in children undergoing abdominal irradiation.

## Patients and methods

We identified twenty children successively treated in our institution from 2005 to 2012 who had received at least 10 Gy of RT to the abdomen, for conditions such as abdominal cancer, nephroblastoma, neuroblastoma, Hodgkin lymphoma requiring splenic and abdominal irradiation, or medulloblastoma requiring craniospinal irradiation.

All patients underwent computed tomography (CT) simulation in their treatment position with personalized immobilization device using a Brilliance CT Big Bore scanner (Philips, The Netherlands). The planning volume was scanned in 3-mm increments without contrast injection. 4D CT was not used. Clinical target volumes (CTV) were determined by a physician expert in paediatric radiation oncology as follows: for medulloblastoma: whole brain and spinal axis; nephroblastoma: nephrectomy bed and para-aortic lymph nodes from the renal arteries to the aortic bifurcation; neuroblastoma: initial tumor site; Hodgkin lymphoma: spleen and para-aortic lymph nodes from the renal arteries to the aortic bifurcation. Planning target volumes (PTV) were automatically delineated by adding an 3D margin to the CTV: 10 mm for medulloblastoma; 5 mm for nephroblastoma and neuroblastoma; for Hodgkin lymphoma: 10 mm to spleen (except for supero-inferior axis: 15 mm), and 5 mm to para-aortic lymph nodes [15]. The abdominal organs at risk were the whole pancreas (WP) and its tail (PT), kidneys, liver, vertebrae, and spinal cord. PT was defined as the part of the organ located at the left border of aorta [16]. The delineation of WP and PT was reviewed by a radiologist expert in abdominal malignancy.

Treatment plans were calculated using Xio (Elekta AB, Stockholm, Sweden) for CRT, Tomotherapy planning system with VoLo (Tomotherapy Incorporated, Madison, WI, USA) for HT and RayStation (Pencil beam algorithm, RaySearch Laboratories, Stockholm, Sweden) for PBT (with beam data of a Proteus plus, IBA, Louvain-La-Neuve, Belgium). Both algorithms used for CRT and HT treatment plans were based on collapsed cone dose calculation method. The dosimetric comparison was performed with Artiview (Aquilab, Lille, France). Prescribed doses were 36 Gy in 36 fractions for medulloblastoma, 14.4 Gy and 10.8 Gy boost to para-aortic lymph nodes in 14 fractions for nephroblastoma, 21 Gy in 14 fractions for neuroblastoma, and 19.8 Gy in 11 fractions for Hodgkin lymphoma, considering a Relative Biological Effectiveness of 1.1 for protons. The PTV was planned to receive at least 95% of the prescribed dose. CRT was performed with multiple 6 and/or 23 MV photon beams. For HT, the field width used for treatment planning and optimization was 2.5 cm. The pitch was 0.430 for medulloblastoma, and 0.287 for other locations. Modulation factors ranged from 2.2 to 2.8. All protons plans are IMPT plans with 3 mm spot and an energy layer of 5 mm, optimization were performed with multiple beams, without robustness for the range incertitude with the same coverage goal for PTV. As robust optimization is not available for all techniques in our institution, the method proposed in current work provides an alternative to compare proton with photon dose distribution. The PBT dose distribution is theoretically the best achievable with a reduce range uncertainty, maybe available with modern imaging systems. Constraints to OAR were: single kidney: V20 Gy < 20%; V12 Gy < 100%; bilateral kidney V30 Gy < 66%; liver V20 < 100%. In-field vertebrae were to receive a homogenous dose higher than 18 Gy. The aim of treatment planning was to keep the dose to the pancreas as low as possible while maintaining the same PTV coverage and the same dose constraints to the other OARs.

Dose-volume histograms were calculated for both target volumes and OAR. The parameters used for comparison were: mean dose to the WP and PT, volume of the WP and PT receiving 10 Gy, volume of PTV receiving 95% of the prescribed dose (V95% PTV), mean dose to the liver and the kidneys, volume of the liver and the kidney receiving 12 Gy and 20 Gy (V12 Gy liver, V12 Gy kidney, V20 Gy liver, V20 Gy kidney), volume of tissue receiving 10 and 5 Gy (V10 Gy and V5 Gy). We also calculated and compared integral dose as follows: volume of normal tissue (total volume–CTV volume) times mean dose to normal tissue. In this study on abdominal irradiation, patients were assumed to be of uniform density for integral dose calculation [17]. The conformity index was calculated using the formula: volume of tissue receiving 95% of the prescribed dose / volume of PTV [12]. The homogeneity index was calculated using the formula: (D2% PTV–D98% PTV) / D50% PTV; where D2% PTV is the dose received by 2% of the PTV (near-maximum dose), D98% PTV is the dose received by 98% of the PTV (near-minimum dose) and D50% PTV is the median dose to PTV.

Patients were selected among the PediaRT French database on Pediatric Radiotherapy. Patient information was anonymized to protect patient confidentiality. This study was approved by Commission Nationale de l'Informatique et des Libertés (French: National Commission for Computing and Liberties) and Comité Consultatif sur le Traitement de l'Information en Matière de Recherche dans le Domaine de la Santé (French: Advisory Committee on Information Processing in Material Research in the Field of Health).

Values were compared using Wilcoxon signed-rank test. Statistical analysis was performed with Stata 13.1 Special Edition (StataCorp LP, College Station, Texas 77845 USA). Results were considered significant at the 0.05 level.

## Results

### Patient characteristics

Twenty patients (13 boys, 7 girls) were selected (median age 8, range 1–14), having five medulloblastomas (midline irradiation), three left-sided nephroblastomas, two right-sided nephroblastomas, one left-sided neuroblastoma, four midline or right-sided neuroblastomas, and five Hodgkin lymphomas (midline and left-sided). All patients were actually treated with CRT except for two patients with medulloblastoma treated with HT.

### Dose to target volumes

**Fig 1:**
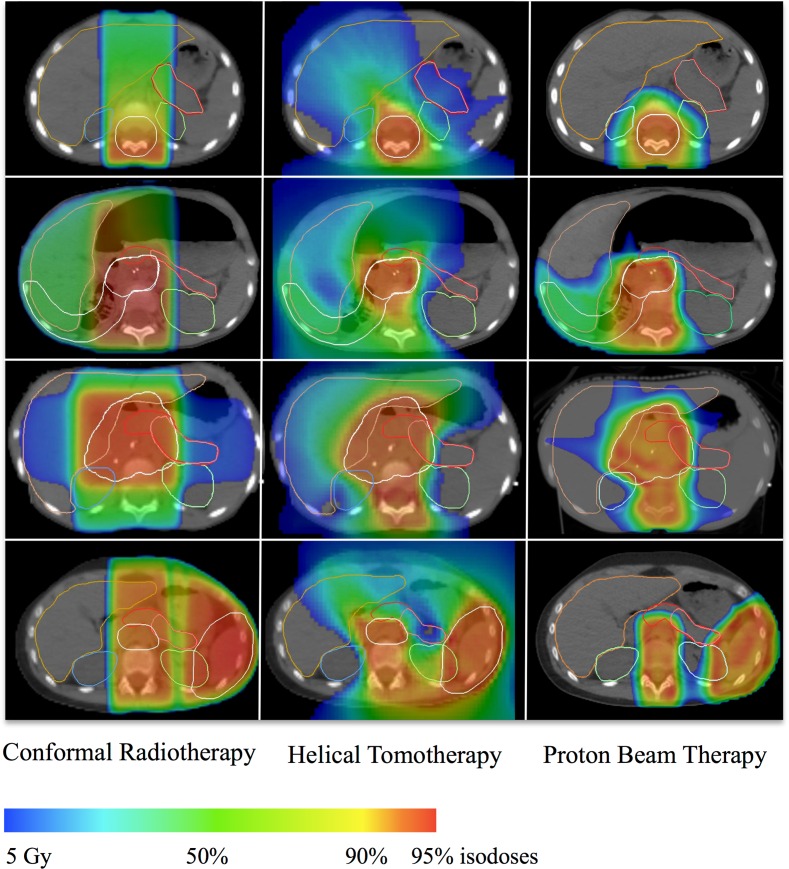
Dose distribution in four illustrative cases. A: medulloblastoma; B: right-sided nephroblastoma; C: median neuroblastoma; D: Hodgkin lymphoma. Left panel: conformal radiotherapy; right panel: helical tomotherapy. PTV contours (white). 95% isodoses (red); 90% isodose (yellow); 50% isodose (green); 5 Gy isodose (blue).

Median PTV volumes for medulloblastoma, nephroblastoma, neuroblastoma and Hodgkin lymphoma were 2231 cm^3^ (range 2099–2410), 153 cm^3^ (range 85–197), 231 cm^3^ (range 67–325), and 686 cm^3^ (range 658–821) respectively. PTV coverage was similar using the three techniques with a mean V95% PTV of 98.01% with CRT, 97.43% with HT, 96.14% with PBT (Table 1). The dose constraint V95% PTV > 95% was achieved in all patients using the three techniques. Conformity indexes were always lower when HT was used compared to CRT except in one patient with median neuroblastoma. When using PBT, conformity indexes were lower in all cases compared to CRT or HT. The mean values were 3.09 (standard deviation [SD] = 1.49) with CRT, 1.65 (SD = 0.51) with HT (p = 0.0002) and 1.36 (SD = 0.50) with PBT (p = 0.0001). Homogeneity indexes were not different between CRT and HT while they were significantly higher with PBT. Dose distributions in four relevant cases are presented in Fig 1.

**Table 1:** Dose to Target volumes.

	CRT	HT	PT	p		
				CRT vs. HT	CRT vs. PT	HT vs. PT
V95% PTV (%) (range)	98.01 (95–100)	97.43 (95–100)	96.41 (95–100)	0.31	0.0003	0.01
Conformity index (SD)	3.09 (1.49)	1.65 (0.51)	1.37 (0.50)	0.0002	0.0001	0.0001
Homogeneity index (SD)	0.11 (0.06)	0.08 (0.03)	0.18 (0.06)	0.17	0.01	0.001

CRT: conformal radiotherapy; HT: helical tomotherapy; SD: standard deviation

### Dose to the pancreas and pancreatic tail

Globally, mean dose and V10 Gy to the WP and especially to the PT were reduced using HT compared to CRT (Table 2). Doses were even more reduced when using PBT. Mean dose to the PT was 16.72 Gy (SD = 5.82) with CRT, 10.72 Gy (SD = 4.99) with HT (p = 0.0001) and 6.63 (SD = 6.45) with PBT (p = 0.0001); V10 Gy to the PT was 75% (SD = 30.11) with CRT, 46% (SD = 35.2) with HT (p = 0.0002) and 29% (SD = 33.02) with PT (p = 0.0001).

**Table 2:** Dose to the whole pancreas and to the pancreatic tail in all cases and by tumor location

	CRT	HT	PT	p		
				CRT vs. HT	CRT vs. PT	HT vs. PT
**V10 Gy (%) (SD)**						
Pancreas	85.75 (18.08)	57.47 (29.39)	38.30 (31.59)	0.0001	0.0001	0.0001
Pancreatic tail	74.69 (30.11)	45.46 (35.20)	28.76 (33.02)	0.0002	0.0001	0.0003
**Mean dose (Gy) (SD)**						
Pancreas	19.67 (4.03)	12.56 (5.05)	8.60 (6.57)	0.0001	0.0001	0.0001
Pancreatic tail						
All cases (n = 20)	16.72 (5.82)	10.72 (4.99)	6.63 (6.45)	0.0001	0.0001	0.0001
Left-sided (n = 4)	22.11 (2.14)	17.72 (4.44)	14.18 (7.02)	0.0679	0.0679	0.14
Midline/right-sided (n = 11)	13.34 (5.80)	8.02 (3.62)	3.34 (5.30)	0.0058	0.0033	0.0033
Midline/left-sided (n = 9)	20.85 (1.81)	14.03 (4.52)	10.66 (5.52)	0.0077	0.0077	0.01
Midline (n = 12)	17.02 (5.60)	9.29 (3.77)	5.69 (5.29)	0.0037	0.0022	0.0022

CRT: conformal radiotherapy; HT: helical tomotherapy; SD: standard deviation

Midline/right-sided tumors: medulloblastomas, right‐sided nephroblastomas and right‐sided and median neuroblastomas. Midline/left-sided tumors: lymphomas, left-sided nephroblastomas and neuroblastomas. Midline tumors: medulloblastomas, median neuroblastomas, lymphomas

When looking according to tumor location, mean dose to the PT was significantly reduced with HT and PBT compared to CRT in midline and right-sided tumors, but not in left-sided tumor; doses were significantly even lower in these tumors with PT compared to HT.

Mean dose to the PT was reduced by more than 10% in 17 of the 20 patients with HT (median reduction: 28%; range: 0–80%) and 18 patients with PBT (median reduction: 67%; range: 1–98%).

### Dose to other OAR and normal tissue

V12 Gy and V20 Gy of the liver (n = 20), contralateral kidney (n = 13) and ipsilateral kidney (n = 8) were reduced with HT compared to CRT, but a significant reduction on the mean dose was only seen for the ipsilateral kidney. Using PBT decreased V12 Gy,V20 Gy and mean dose in all considered OAR compared to CRT. While V12 Gy,V20 Gy and mean dose of the liver were reduced with PBT compared to HT, the dose reduction between these two techniques on the contra- or ipsilateral kidneys did not reach significance. Integral dose and V10 Gy did not differ significantly between HT and CRT, but V5 Gy was increased with the use of HT. Irradiation of normal tissue in terms of integral dose, V10 Gy, V5 Gy was decreased using PBT compared to both CRT and HT (Table 3).

**Table 3:** Dose to OARs and normal tissue.

	CRT	HT	PT	p		
				CRT vs. HT	CRT vs. PT	HT vs. PT
**Liver**						
V12 Gy (%) (SD)	42.82 (26.50)	23.53 (11.74)	6.82 (9.41)	0.0001	0.0001	0.0001
V20 Gy (%) (SD)	22.33 (15.27)	4.0 (6.34)	2.76 (5.52)	0.0001	0.0001	0.0014
Mean dose (Gy) (SD)	9.24 (4.68)	8.95 (2.06)	2.57 (2.62)	0.58	0.0001	0.0001
**Contralateral kidney**						
V12 Gy (%) (SD)	13.0 (11.30)	2.72 (8.57)	3.60 (6.09)	0.013	0.0019	0.40
V20 Gy (%) (SD)	5.33 (8.18)	0.00 (0.01)	0.43 (1.18)	0.0266	0.0266	0.0266
Mean dose (Gy) (SD)	4.59 (2.70)	3.13 (0.54)	2.33 (1.96)	0.087	0.013	0.13
**Ipsilateral kidney**						
V12 Gy (%) (SD)	63.50 (13.91)	47.22 (18.49)	41.43 (7.39)	0.025	0.0117	0.67
V20 Gy (%) (SD)	42.85 (14.13)	15.97 (14.53)	19.94 (6.45)	0.012	0.012	0.40
Mean dose (Gy) (SD)	13.82 (2.76)	10.64 (4.22)	10.51 (1.21)	0.017	0.017	0.67
**Dose to normal tissue**						
Integral dose (Gy.L) (SD)	72.51 (50.76)	72.73 (44.92)	43.0 (26.09)	0.19	0.0001	0.0001
V10 Gy (L) (SD)	3.41 (2.12)	3.32 (2.14)	2.11 (1.56)	0.33	0.0001	0.0001
V5 Gy (L) (SD)	3.84 (2.24)	6.36 (4.87)	2.60 (1.70)	0.0001	0.0001	0.0001

CRT: conformal radiotherapy; HT: helical tomotherapy; SD: standard deviation

## Discussion

To decrease the dose to the abdominal organs at risk, we compared dosimetric plans using conformal radiotherapy (CRT), helical tomotherapy (HT) and proton beam therapy (PBT) in 20 children undergoing abdominal irradiation. Our data suggest that both HT and PBT could reduce the dose to the pancreas and its tail compared to CRT, especially in right-sided or midline tumors. PBT decreased even more the dose to the pancreas compared to HT in these cases. While the three techniques were able to satisfactorily cover the target volume, PBT offered superior conformity, provided better sparing of the other OAR, and permits to decrease low-dose irradiation to normal tissue.

De Vathaire et al. showed that the risk of DM increased strongly with radiation dose to the PT with an increase in DM risk per Gray of 0.66 (95% CI 0.23–1.71). Here we show that HT and PBT reduce the dose to the pancreas. Mean dose to the PT was reduced by 31% on average with HT and 61% with PBT from 8 Gy in left-sided tumors to almost 12 Gy in midline tumors. According to De Vathaire et al., this reduction would lead to a mean decrease of 20% and 40% in the relative risk of DM with HT and PBT respectively (a reduction of 16 to 67% depending on tumor location with PBT). Considering the pancreas as an OAR and using IMRT or PBT for abdominal irradiation could reduce the incidence of DM among childhood cancer survivors, so the pancreas should be considered a critical organ during RT. A recent dosimetric analysis on five patients treated for medulloblastomas also reported significant reductions of mean dose and V10 Gy to the WP and PT using HT; reductions were also even higher with PBT; dose to others OAR and normal tissue were not studied [18].

In this study, decreasing the dose to the pancreas did not affect PTV coverage or increase the radiation dose received by other abdominal OAR. Good target coverage that exploits the superior conformity and homogeneity offered by new RT techniques may assure a better tumor control with an improved dose-to-tumor distribution [19]. To achieve good coverage with CRT, the volume receiving high dose must be large: in our study the conformity index with CRT was approximately twice that with HT and beyond compared to PBT. Other reports have shown similar results [12, 13, 20]. The mean dose received was considerably reduced with HT in the ipsilateral kidney thanks to the reduction of volume that received a high dose. While HT failed to decreased mean dose to the contralateral kidney and the liver because of low-dose bath, PBT showed better dosimetric results in all considered OAR. Similarly, other studies showed a benefit of IMRT/HT or PBT as compared to CRT in terms of dose to the abdominal OAR in neuroblastoma [12], nephroblastoma [13].

HT increased the volume of normal tissue receiving 5 Gy. At the contrary, PBT permitted to decrease the radiation dose to normal tissue compared to CRT and HT. One crucial argument against the use of IMRT and HT in children, and particularly younger children, has been the increase in low doses received outside the target with these techniques [21]. While ionizing radiation has been recognized as carcinogenic for many years, the carcinogenic effect of low doses is not completely understood [22]. The risk of secondary malignancies has been showed to be related to the integral dose [23] or the volume of the intermediate-dose region [24]. In the present study, the integral dose and the volume of normal tissue receiving more than 10 Gy (V10 Gy) did not significantly differ between HT and CRT. As in other published studies [12, 13], V5 Gy was increased, which may be explained by the increase in entrance and exit dose due to the increased number of beams required by IMRT. The predicted doubling of incidence of secondary malignancies using IMRT [25] warrants careful decision-making when considering IMRT for children with malignancies and may be an argument in favor of the use of PBT. Nevertheless secondary neutrons produced during PBT may severely increase the risk of secondary malignancy and should be kept in mind [13]. Longer term follow-up data is needed for these relatively recent techniques.

One limitation of our study is that the pancreas was delineated on simulation CT scans without contrast agent. Distinguishing between pancreatic tissue and bowel is particularly difficult in children because of the paucity of retroperitoneal fat. A simulation CT scan with intravenous contrast agent or in combination with abdominal MRI might be advisable for treatment planning as these modalities are considered the best way to visualize the pancreas [26]. Another limitation is the mobility of the abdominal OAR. The mobility of the kidneys is well-recognized [27], but data on the mobility of the pancreas (and especially in children) are lacking. In adults, different parts of the pancreas move differently, with the tail moving most of all [28, 29]. The average displacement of the pancreas was measured at 18 to 20 mm, with the greatest displacement in the supero-inferior axis, mainly due to diaphragmatic movement [28, 30]. An individualized organ motion study may be necessary [31]. Both organ and target motion is a key issue in IMRT/PBT and image-guided radiation therapy to the upper abdomen. In a study of eight children with high-risk neuroblastoma, IMRT planning with volume definition based on image guidance (4-D CT and MRI fusion) enabled volumetric reduction of the PTV and a reduced dose to normal tissue compared to IMRT planning with conventional volumetric construction [32].

## Conclusion

This study is the largest dosimetric comparison of CRT, HT and PBT in children undergoing abdominal irradiation in terms of the dose received by the pancreas and other abdominal OAR. Both HT and PBT allowed reducing the dose to the pancreas and ipsilateral kidney while maintaining good PTV coverage, especially in midline and right-sided tumors in comparison with CRT. PBT increased even more the reduction of dose to the pancreas compared to HT and reduced the irradiation of contralateral kidney, liver and normal tissue while low doses were increased with the use of HT. The potential dosimetric benefits of IMRT techniques and proton beam therapy compared with conformal radiotherapy should therefore be considered for each child undergoing radiotherapy for abdominal malignancies in order to reduce the risk of late abdominal toxicity including diabetes mellitus.
